# Effects of Inflammation on Biomarkers of Vitamin A Status among a Cohort of Bolivian Infants

**DOI:** 10.3390/nu10091240

**Published:** 2018-09-05

**Authors:** Rachel M. Burke, Ralph D. Whitehead, Janet Figueroa, Denis Whelan, Anna M. Aceituno, Paulina A. Rebolledo, Rita Revollo, Juan S. Leon, Parminder S. Suchdev

**Affiliations:** 1 Department of Epidemiology, Rollins School of Public Health, Emory University, Atlanta, GA 30322, USA; janet.figueroa@emory.edu; 2 Nutrition Branch, Division of Nutrition, Physical Activity, and Obesity, Centers for Disease Control & Prevention, Atlanta, GA 30341, USA; rnw2@cdc.gov (R.D.W.J.); psuchde@emory.edu (P.S.S.); 3 Department of Biostatistics, Rollins School of Public Health, Emory University, Atlanta, GA 30322, USA; denis.r.whelan@gmail.com; 4 Hubert Department of Global Health, Rollins School of Public Health, Emory University, Atlanta, GA 30322, USA; anna.m.aceituno@gmail.com (A.M.A.); preboll@emory.edu (P.A.R.); juan.leon@emory.edu (J.S.L.); 5 Department of Pediatrics, Emory School of Medicine, Atlanta, GA 30322, USA; 6 Servicio Departamental de Salud, La Paz, Bolivia; ritarevollom@hotmail.com

**Keywords:** vitamin A deficiency, infant nutrition, micronutrient deficiencies, global micronutrient malnutrition

## Abstract

Globally, vitamin A deficiency (VAD) affects nearly 200 million children with negative health consequences. VAD can be measured by a retinol-binding protein (RBP) and serum retinol concentrations. Their concentrations are not always present in a 1:1 molar ratio and are affected by inflammation. This study sought to quantify VAD and its impact on infant mortality and infectious morbidity during the first 18 months of life in a cohort of mother-infant dyads in El Alto, Bolivia, while accounting for the previously mentioned measurement issues. Healthy mother-infant dyads (*n* = 461) were enrolled from two hospitals and followed for 12 to 18 months. Three serum samples were collected (at one to two, six to eight, and 12 to 18 months of infant age) and analyzed for RBP, and a random 10% subsample was analyzed for retinol. Linear regression of RBP on retinol was used to generate RBP cut-offs equivalent to retinol <0.7 µmol/L. All measures of RBP and retinol were adjusted for inflammation, which was measured by a C-reactive protein and alpha (1)-acid glycoprotein serum concentrations using linear regression. Infant mortality and morbidity rates were calculated and compared by early VAD status at two months of age. Retinol and RBP were weakly affected by inflammation. This association varied with infant age. Estimated VAD (RBP < 0.7 µmol/L) decreased from 71.0% to 14.8% to 7.7% at two, six to eight, and 12 to 18 months of age. VAD was almost nonexistent in mothers. Early VAD was not significantly associated with infant mortality or morbidity rates. This study confirmed a relationship between inflammation and vitamin A biomarkers for some subsets of the population and suggested that the vitamin A status in early infancy improves with age and may not have significantly affected morbidity in this population of healthy infants.

## 1. Introduction

Vitamin A deficiency (VAD) affects an estimated 190 million children under five years of age worldwide with the greatest burden in low-income and middle-income countries (LMIC) [1]. Numerous critical biological functions are affected by vitamin A and deficiency can lead to increased susceptibility to infections, night blindness, and even full vision loss [1]. Given the potentially severe consequences of VAD, many countries have implemented supplementation and fortification programs and the prevalence of VAD has declined over time [2]. Nonetheless, in many areas, VAD persists as a public health problem while, in other areas, data on VAD prevalence is outdated or does not exist at the national level [3,4].

Although vitamin A liver stores are the gold standard for the measurement of vitamin A status, it is more common to use serum retinol or its carrier known as the retinol binding protein (RBP) as a surrogate given the invasiveness of the liver biopsy and the time and resources required for relative dose response tests to assess vitamin A total body stores [5,6]. Retinol levels <0.7 µmol/L are commonly considered as representative of VAD in adults and children ≥6 months of age, and the prevalence of individuals with serum retinol <0.7 µmol/L or RBP concentrations <0.7 µmol/L is used by the World Health Organization to assess population VAD and to reflect the severity of VAD as a public health problem [1,7]. RBP is considered to be similar in molar ratio (1:1) as retinol, which requires less volume of blood to assay and is less expensive to measure [5,8]. Nonetheless, the ratio of retinol to RBP may be affected by extraneous factors such as vitamin A status and inflammation, as well as a nutritional status especially obesity [7,9,10]. Thus, it is important to evaluate this relationship in multiple populations. Specifically, when a linear model is used to compare retinol to RBP in a population where both are measured, correlations have ranged from 0.50 to 0.95, and molar ratios have been both above and below one [11,12,13,14,15,16]. Furthermore, RBP and retinol are themselves affected by the acute phase response (APR), which is the body’s inflammatory reaction to trauma, infection, or stress [17,18]. Specifically, both retinol and RBP have been shown to be depressed by as much as 40% in the presence of inflammation—whether caused by trauma or infection. However, this relationship was recently observed to be stronger in preschool children when compared to women [17,19,20,21]. Acute phase proteins (APP) can be used to adjust retinol and RBP for the inflammatory effect, facilitating a more accurate interpretation of a vitamin A status. The APPs C-reactive protein (CRP) and alpha(1)-acid glycoprotein (AGP) are commonly used for this purpose [10,17,22].

Young infants are at higher risk of low vitamin A status than adults. Although fetuses accumulate hepatic stores of vitamin A during the last trimester of pregnancy, infants are typically born with half the circulating serum retinol as their mothers [23]. Because retinol can be teratogenic, women do not receive high-dose supplementation during pregnancy. However, supplementation is common in the immediate postpartum setting particularly because retinol is transferred into breastmilk through which infants receive Vitamin A [24]. Infants, however, do not typically receive vitamin A supplementation (VAS) until six months of age since systematic reviews have not indicated any morbidity or mortality benefit of VAS before this time [25,26,27]. Few studies exist that longitudinally measure vitamin A status between birth and 6 months of age, and the biomarker cut-offs used to assess vitamin A status in older infants and children are not fully validated in infants below the age of 6 months. Consequently, little is known about the evolution of the vitamin A status, the development and prevalence of VAD, or the potential health effects of low vitamin A in healthy, exclusively breastfeeding infants before the initiation of biannual supplementation.

The primary aim of the present study is to describe the vitamin A status in a cohort of mother-infant dyads in El Alto, Bolivia. Specifically, we wished to explore the impact of inflammation on RBP and retinol and to examine the relationship between these two biomarkers. A secondary objective was to assess whether a low vitamin A status at two months of age (RBP < 0.7 μmol/L) had any measurable impact on later infant mortality and rates of infectious morbidity. A tertiary objective was to identify correlations of infant VAD to guide the potential impact of vitamin A programs. This study will provide useful information from a population of individuals of primarily indigenous descent residing at a high altitude in the setting of national micronutrient supplementation programs. 

## 2. Methods

### 2.1. Study Population and Design

Data for the present study were drawn from the *Nutrición, Inmunología, y Diarrea Infantil* (NIDI) study, which had a primary aim of assessing the effect of an infant nutritional status to respond to the rotavirus vaccine (scheduled at 2 and 4 months of age). The study setting of El Alto (altitude 4000 m) is a peri-urban locale home to a largely indigenous population in which most have relatively few socioeconomic resources. As part of other maternal and child health programming, Bolivian policies recommend vitamin A supplementation (VAS) to mothers immediately postpartum and to infants every 6 months during the 6–59-month age range. Policies also provide for 60 sachets of multiple micronutrient powder (MNP, “Chispitas”, 12.5 g iron as ferrous fumarate, 5 mg zinc as zinc gluconate, 300 µg vitamin A as retinol acetate, 30 mg vitamin C, 180 µg folic acid, intended for usage at 1 sachet/day), which is provided on the same twice-yearly schedule as child VAS [28]. Vitamin A fortification of vegetable oil has been mandated in Bolivia since 2005, but not all neighboring countries share this mandate, so levels vary for imported cooking oil. The brand of cooking oil used by participants was not captured in the present study.

Study methods are described in detail elsewhere [29,30]. In brief, 461 healthy infants (2–4 weeks of age) and their mothers were recruited from 2 hospitals during well-child or vaccination visits. Infants were excluded in cases when immunodeficiency, congenital malformations, or acute illness were suspected at the time of enrollment. Inclusion criteria included the maternal ability to speak and understand Spanish or Aymara. Recruitment took place from May 2013 through March 2014. Participants were followed for 12 to 18 months and final data were collected in March 2015. For any infant who died during the period of study participation, a verbal autopsy was conducted to rule out the relationship for the study activities.

The study comprised 7 hospital visits and 2 in-home visits. At each study visit, feeding and supplementation practices were queried and mothers were asked whether their child had experienced fever, respiratory illness/cough, diarrhea, or any other illness within the prior two weeks. Mothers were also instructed to call the study staff if their child developed diarrhea between study visits. A morbidity episode was defined as a report of one or more of these symptoms including diarrhea reported between study visits. The exclusive breastfeeding status was based on a maternal recall of feeding practices through the most recent visit. Exclusive breastfeeding was defined as a maternal report of feeding the child only breast milk without any non-breast milk liquids or solid or semi-solid foods.

Blood was drawn from mothers at a target schedule of 1 and 6 to 8 months postpartum and from infants at a target schedule of 2 months, 6 to 8 months, and (optionally) 12 to 18 months of age. The third infant blood draw, added to support a newly funded secondary aim, was approved only after 50% of infants had already completed the study. These mothers were contacted to participate optionally in an extra visit and a blood draw. At this time, mothers were also offered anemia testing for themselves via a finger stick. Infants completing three blood draws were not meaningfully different from those whose mothers did not consent to the third blood draw.

For the primary aim of assessing infants’ vitamin A status, all singleton infants with at least the first blood draw were included for each time point where they had available data. For the secondary aim of assessing potential consequences of early VAD (i.e., VAD at 2 months of age), the analytical population included only singletons with the first blood draw who were followed until the time of the second blood draw. Inclusion was not dependent on the success of the second blood draw. Mothers were included at each time point based on whether they had available data.

### 2.2. Ethical Approval

The protocol and instruments for this study were approved by the Emory University IRB (IRB00056127) and the Bolivian Comité de Etica de la Investigación (Research Ethics Committee). Mothers provided written informed consent in Spanish or Aymara after demonstrating an understanding of the study goal and requirements.

Mothers and infants were referred to hospital clinicians for anemia, according to Bolivian guidelines (hemoglobin < 13.7 g/dL for mothers, hemoglobin < 10.9 g/dL for infants and toddlers, with no altitude adjustment) [28]. Infants were also referred for stunting (length-for-age *Z* score < −2) or wasting (weight-for-length *Z* score < −2) as diagnosed by trained anthropometrists based on WHO growth charts [31].

### 2.3. Laboratory Analysis

Venous blood was collected (1 mL) from mothers and infants using zinc-free syringes and tubes. Hemoglobin (Hb) was measured at point-of-care using a HemoCue^®^ Hb-201^+^ photometer. Plasma was analyzed at the VitMin Lab by a sandwich ELISA for CRP (a marker of acute inflammation, limit of detection [LOD]: 0.5 mg/L), AGP (a marker of chronic inflammation, LOD: 0.1 g/L), retinol binding protein (RBP, LOD: 0.05 µmol/L), ferritin (Fer, LOD: 2 µg/L), and soluble transferrin receptor (sTFR, LOD: 0.5 mg/L, Ramco assay equivalents, VitMin Lab, Willstaett, Germany) [8].

Given that retinol and RBP are not necessarily present in a 1:1 molar ratio, a random 10% subsample (*N* = 172) of serum aliquots was selected for additional retinol testing. The subsample was spread across mothers, infants, and all time points (*N* = 1736). Retinol was analyzed by high-performance liquid chromatography (HPLC) by the Instituto de Nutrición de Centroamérica y Panamá (INCAP) laboratory (Guatemala City, Guatemala). A retinol acetate internal standard was used in each analytical run. Quality control (QC) samples in 3 levels (low, medium, and high) were used in each analytical run. The inter-assay coefficient of variability (CV) was <10%. Serum control samples (Liquicheck, Bio-Rad, Hercules, CA, USA) were used as standards for the calibration curve. QC samples in two levels (low and high) were used in each analytical run. An inter-assay CV was <4% for RBP. A CV of about 10% provides acceptable precision using an ELISA technique [8]. These data indicate that the lab’s performance exceeded the acceptable performance expectations while analyzing the survey specimens.

### 2.4. Definitions of Anemia, Inflammation, and Vitamin A Deficiency (VAD)

Hb cut-offs for anemia were adjusted for the high altitude (3500 to 4000 m) of El Alto and the surrounding area of La Paz [32]. Anemia was defined as Hb < 13.7 g/dL for infants and as Hb < 14.7 g/dL for mothers (14.4 g/dL for the 10 mothers who reported currently smoking, two mothers who were pregnant at the last visit were excluded from analysis) based on WHO guidelines [33]. Inflammation was defined as CRP > 5 mg/L or AGP > 1 g/L [17,34].

Serum retinol < 0.70 µmol/L is recommended to indicate populations at risk of VAD [1,35]. Given that it is cheaper to measure, RBP is often used in a 1:1 molar ratio as a surrogate marker for retinol. However, since RBP and retinol are not always present in a 1:1 molar ratio, we used linear regression of RBP on retinol to determine the RBP equivalent of a retinol cutoff of <0.70 µmol/L. Using the estimated parameters from this linear model, we solved the regression equation to determine the RBP equivalent of 0.70 µmol/L retinol among infants and mothers. This regression did not take into account the inflammation status of the individuals. The regression-calculated RBP cut-off was <0.33 µmol/L for infants and <0.65 µmol/L for mothers. In the present analysis, we compared the VAD prevalence using three different definitions: (1) serum retinol < 0.70 µmol/L, (2) serum RBP < 0.70 µmol/L, and (3) serum RBP < 0.33 µmol/L (infants)/< 0.65 µmol/L (mothers). Through this method, we tested the impact of assuming a 1:1 molar ratio for RBP to retinol given that this common assumption was not valid for our population. Since a validated cutoff for VAD does not exist in children <6 months of age, we applied the cutoff of RBP < 0.70 μmol/L to classify these infants for the purposes of analyses of “low Vitamin A status in early infancy”.

### 2.5. Adjustment of Vitamin A Biomarkers for Inflammation

Given the well-described effects of inflammation on RBP and retinol, we adjusted the biomarkers’ concentrations for CRP and AGP [17]. Based on the Biomarkers Reflecting Inflammation and Nutrition Determinants of Anemia (BRINDA) project [36] and other publications [22,37], we employed a correction method based on linear regression since it best accounts for the linear (or log-linear) relationship between both CRP and AGP and RBP and, therefore, does not rely on arbitrary CRP and AGP cutoffs [37]. For the body of the present paper, we present the crude (unadjusted) and adjusted results. For each population subset (2-month-old infants, 6–18-month-old infants, mothers), the vitamin A biomarker, i.e., RBP or retinol, (log-transformed to meet normality assumptions) was modeled as a function of continuous CRP and AGP (also log-transformed to improve the model fit).

(1) $$\;\ln\left(\mathrm{RBP}\right)=\beta_{0}+\beta_{1}\ln\left(\mathrm{CRP}\right)+\beta_{2}\ln\left(\mathrm{AGP}\right)\;$$

In the above equation, the intercept (β_0_) represents the natural log of the counterfactual RBP that would have been observed in the absence of inflammation. This equation is then solved for this quantity, which is hereby denoted as ln (RBP_corr_) to show that it is the estimated “inflammation-corrected” value of the natural log of RBP. Similarly, we must account for the CRP and AGP that are above the “normal” values. We do so by subtracting the chosen reference values from the observed values.

(2) $$\;\ln\left({\mathrm{RBP}}_{\mathrm{corr}}\right)=\ln({\mathrm{RBP}}_{\mathrm{obs}})-{\hat{\hat{\beta }}}_{1}\left(\ln\left({\mathrm{CRP}}_{\mathrm{obs}}\right)-\ln\left({\mathrm{CRP}}_{\mathrm{ref}}\right)\right)-{\hat{\hat{\beta }}}_{2}\left(\ln\left({\mathrm{AGP}}_{\mathrm{obs}}\right)-\ln\left({\mathrm{AGP}}_{\mathrm{ref}}\right)\right)\;$$

Since there are no established reference values for normal CRP and AGP in the literature, the first deciles of CRP and AGP in the populations were used as the reference values for the reported results. This approach is consistent with decisions made by Larson et al. [19]. Given potential differences in vitamin A metabolism as well as the acute phase response and, based on examination of the data, mothers (1st deciles 0.44 mg/L CRP, 0.49 g/L AGP) were adjusted separately from infants (1st deciles 0.14 mg/L CRP, 0.31 g/L AGP). The RBP and retinol values for the youngest infants were not adjusted at all since no relationship was visually observed between inflammation and vitamin A biomarkers. Data from these infants were also excluded from the data used to generate regression equations for older infants.

### 2.6. Statistical Methods

The relationships among RBP, retinol, AGP, and CRP were plotted and visually examined. RBP and VAD were also plotted by the quintile of AGP and CRP to inform decisions on the adjustment for inflammation. The Cochran-Armitage test for the trend was used to analyze whether the proportion of uncorrected VAD varied by the APP quintile. To assess differences between continuous variables (e.g., RBP) at different time points, the Wilcoxon signed-rank test was used given non-normal distributions and paired data. To assess differences between categorical variables at different time points, McNemar’s test for paired nominal data was used. Fisher’s exact test was used to test unpaired categorical data. For the secondary objective of determining potential health consequences of low vitamin A status in infants <6 months of age, the rates of morbidity episodes (report of cough, fever, diarrhea, or other illness) between the first (2 months of age) and second (~6–8 months of age) blood draws were compared between children VAD vs. non-VAD at the first blood draw (defined as RBP <0.70 µmol/L). Morbidity rates were calculated for each child using the total number of unique morbidity episodes reported by the child’s mother between the first and second blood draws over the total time in the study between these two blood draws. As a secondary analysis, rates were also compared for the time period between the first blood draw and the child’s final study visit. Poisson models were used to compare morbidity rates between the two groups (modeling episode counts with an offset of log (time on study) to calculate rate ratios and adjusting a priori for low birth weight (<2500 g) as well as adjusting for maternal employment as a marker of socioeconomic status and inflammation-adjusted ferritin (as above) as a marker of iron status. All variables were retained in each model (primary analysis: morbidity rates between the first and second blood draws, secondary analysis: morbidity rates between the first blood draw and the final study visit). Crude mortality rates were also calculated and compared for infants VAD at the first blood draw versus infants non-VAD at the first blood draw by again utilizing the time period between the first and second blood draws (i.e., 2 to 6 months of age, approximately). However, a post-hoc power analysis demonstrated low power to detect differences in mortality (<10%) given the low number of deaths.

For the tertiary objective of identifying potential correlates of VAD in early life (as defined by crude RBP <0.70 µmol/L at 2 months of age), logistic regression models for determining the vitamin A status at the first blood draw were constructed using the following predictors selected a priori based on literature review: infant age, exclusive breastfeeding, preterm birth, low birthweight, sex, infant stunting, infant overweight or obesity, infant ferritin (a marker of iron status), maternal age, maternal employment, maternal education, maternal self-identified ethnicity (indigenous vs. non-indigenous), and maternal overweight. Since this was an exploratory exercise, the model was reduced using backwards elimination based on the significance of effects with an alpha level of 0.05. Few data (~15%) were missing. Cases with missing data on outcomes or predictors of interest were excluded from models. Only data from the time point of the first blood draw were used in this analysis. Data were cleaned (e.g., examination of outliers, exclusion of biologically improbable or logically inconsistent values, and creation of new variables) and analyzed using SAS v9.4 (Cary, NC, USA) and the R Environment for Statistical Computing [38].

## 3. Results

### 3.1. Characteristics of the Study Population

The study population for the analysis of VAD prevalence included 365 singleton infants with data from the first blood draw. A total of 310 infants (85%) had data from the second blood draw, and 168 infants (46%) had data from the third blood draw. Five twin pairs were excluded from analysis, 64 singleton infants were lost to follow up or excluded for failure to adhere to the vaccination schedule, and 24 singletons were excluded for insufficient or failed blood draw. Among mothers, 455 had data from the first blood draw (which occurred one month prior to the infants’ first blood draw), and 363 infants had data from the second blood draw.

Infants were enrolled at an average age of one month with the first blood draw occurring at an average of 2 months of age (Table 1). The gender distribution was fairly even, and one third of infants were born via Caesarian section, one fifth were preterm, and less than one tenth were of low birth weight. Nearly all infants were breastfed at some point and half were first-born children. The mean maternal age was 25.4 years, and over half of the mothers were overweight or obese. Most mothers had at least a secondary education and nearly all were married or cohabiting with a partner. Socio-demographics varied among the sample, with only a quarter of households owning a refrigerator but over half having access to a private toilet. Approximately three quarters of infants were anemic, while stunting varied from 14% to 21% and overweight factors varied from 19% to 34% (Table 2). Most infants continued breastfeeding through the first year of life. Among mothers, anemia prevalence decreased from the first (30%) to the second (15%) blood draw.

### 3.2. Retinol, Retinol-Binding Protein, and Inflammation

**Figure 1:**
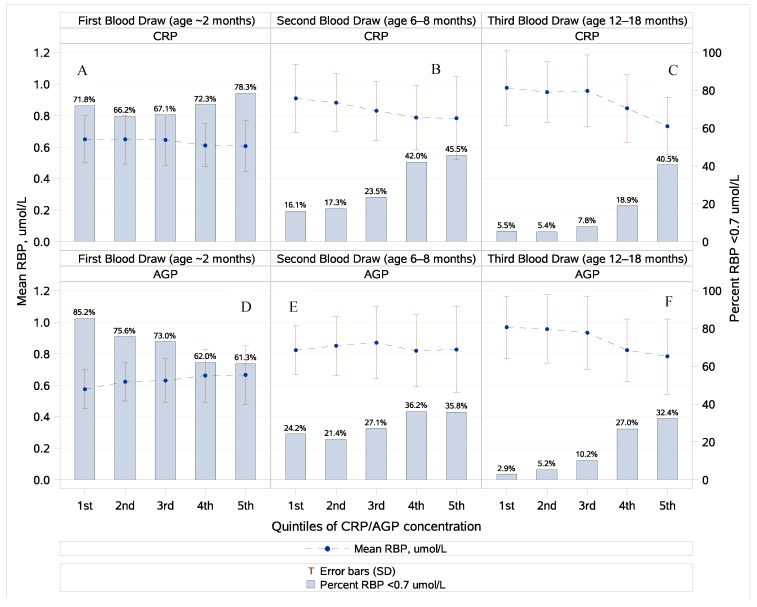
Prevalence of infant vitamin A deficiency (VAD; RBP < 0.70 µmol/L) and mean RBP by quintiles of the C-reactive protein (CRP) and the alpha(1)-acid glycoprotein (AGP) and by blood draw. Panels (**A**–**C**) show VAD by CRP quintiles for blood drawn at ~2, 6–8, and 12–18 months, respectively. Panels (**D**–**F**) show VAD by AGP quintiles for the same time periods. The prevalence of VAD was not significantly different (Cochran Armitage trend test, *p* = 0.23) by CRP quintile for infants measured at the first blood draw (~2 months of age, panel (**A**)) even though VAD seemed to decrease slightly by the AGP quintile at the first blood draw (Cochran Armitage trend test, *p* = 0.0001, panel (**D**)). However, prevalence of VAD significantly increased by CRP and AGP quintile for infants at the second and third blood draws (6–8 and 12–18 months of age).

Both retinol and RBP were weakly affected by the presence of inflammation and the strength and direction of this association varied with infant age. In the youngest infants, a null or slight positive effect was seen while, in older infants, retinol and RBP decreased in the presence of inflammation. Slopes were similar for RBP and retinol as functions of CRP and AGP (data not shown). In the second and third blood draws, the prevalence of VAD as defined by RBP <0.70 µmol/L significantly increased with increasing APP quintiles. No such relationship was seen at the first blood draw (Figure 1).

**Figure 2:**
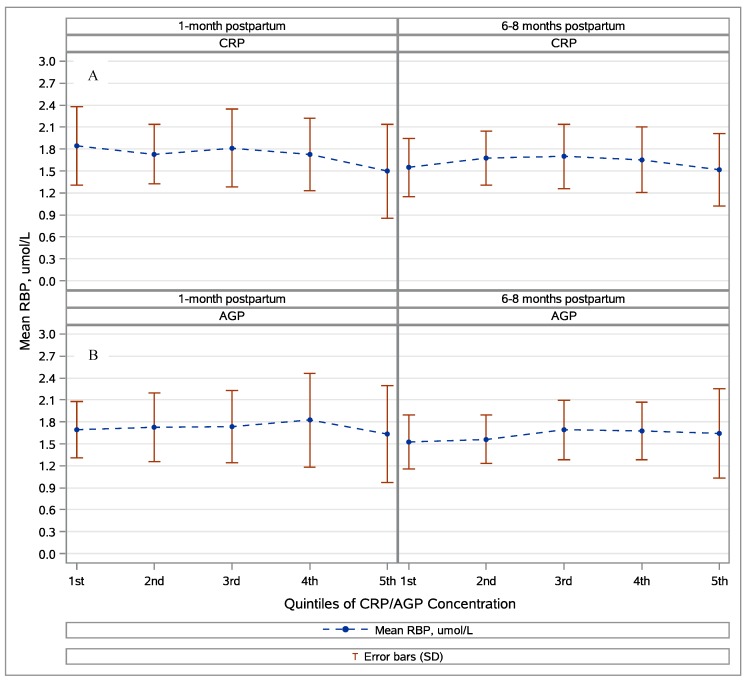
The mean maternal retinol binding protein (RBP) by quintiles of the alpha(1)-acid glycoprotein (AGP) and the C-reactive protein (CRP) by blood draw. Panel (**A**) shows results for the first blood draw (~1 month postpartum) and panel (**B**) shows results for the second blood draw (~6 months postpartum). The mean RBP was not significantly different (Wilcoxon signed rank test, *p* > 0.05) by CRP or AGP quintile for mothers at either blood draw.

In mothers, no clear relationship was observed between retinol and inflammation. There was a small but significant negative relationship of RBP and CRP at the first postpartum visit (β = −0.1, *p* < 0.0001), but RBP was otherwise not significantly associated with inflammation in mothers. There was no relationship between the RBP and the APP quintile (Figure 2).

### 3.3. Vitamin A Status

Estimates of VAD varied depending on the biomarker, the cut-off, the adjustment for inflammation, and the age of the infant (Table 3). However, regardless of the cut-off or biomarker, VAD was consistently highest among infants at 2 months of age and lower in older infants. When using the RBP < 0.33 µmol/L calculated cut-off, no infants were classified as VAD. Adjustment for inflammation tended to increase retinol and RBP concentrations, which reduced estimates of VAD prevalence. Less than 1% of mothers were classified as VAD regardless of the biomarker, the inflammation adjustment, or the time period.

### 3.4. Potential Consequences of Low Vitamin A Status in Early Life

Two of the infants in the analysis population died after the first blood draw. The crude mortality rate among infants who were VAD at 2 months of age was 1.9/1000 child-months as compared to 0/1000 child-months among infants non-VAD at 2 months of age (using unadjusted RBP < 0.70 μmol/L as the definition of VAD). However, the effect was not statistically significant and, due to the low numbers, a rate ratio could not be calculated. The overall morbidity rate was 7.9 episodes (of fever, respiratory illness, or diarrhea) per 100 child-months (Table 4). The morbidity rate for infants classified as VAD at the first blood draw (using unadjusted RBP <0.70 µmol/L) was not significantly different from the rate for infants classified as non-VAD at that blood draw (RR: 0.94, 95% CI: (0.82, 1.09), *p* = 0.41). Secondary analyses using morbidity information up through 12 months of age did not have different conclusions. While the point estimates for the rates were different, the rate ratio was very similar.

### 3.5. Potential Correlations with Low Vitamin A Status in Early Life

Based on logistic regression models for this tertiary objective, only exclusive breastfeeding (aOR: 1.10, 95% CI: (1.01, 1.21), *p* = 0.038) and age (aOR: 0.80 (0.68, 0.95) for a one-month increase, *p* = 0.01) were significantly associated with VAD (as defined by RBP <0.70 µmol/L) at the first blood draw (~2 months of age, *N* = 259/365 (71%)). Anemia was not significantly associated with a vitamin A status at the first blood draw.

## 4. Discussion

The present longitudinal study, which was conducted in a moderate-inflammation setting, demonstrated a weak relationship between inflammation and biomarkers of vitamin A status (e.g., serum retinol and RBP) especially among the maternal population. Among infants 2 months of age, there was a moderate to high prevalence of low vitamin A status when measuring RBP or retinol <0.70 µmol/L, which is a cut-off that has often been used in older populations. Consistent with our current understanding of Vitamin A in infants, the prevalence of VAD sharply decreased as infants aged. However, using the new cut-off generated from the RBP-retinol comparison indicated that VAD was not present at all in these infants at any time point. VAD was virtually non-existent among mothers using any definition. Early-life VAD as defined by 2-month RBP <0.70 µmol/L did not appear to affect the rate of subsequent infectious morbidity episodes among infants. Although both infants who died after the first blood draw had low vitamin A status, this effect was not significant perhaps due to the low sample size. Only infant age (negative association) and exclusive breastfeeding (positive association) were significant predictors of the vitamin A status at the first blood draw (~2 months of age).

In our study population, RBP and retinol were only weakly linearly associated with CRP and AGP especially in mothers. These findings were similar to those in the BRINDA analysis of RBP and APP [19]. For infants in our study population, the associations varied by age at blood draw: there was no clear relationship at 2 months of age, but the expected negative relationship was present at older ages. This may reflect the development of the immune system towards a more pro-inflammatory state [39]. Generally, it may be that linear regression is insufficient to fully characterize the effect of the acute phase response on vitamin A biomarkers. It could also be that our sample size (and majority healthy population) allows for too much influence from outliers. A wider range of inflammatory states may have allowed clearer visualization of a trend [17]. Furthermore, it is possible that the higher prevalence of obesity and overweight in our population (>50% of mothers, >19% in infants) may have modified the inflammatory effect since obesity is a known pro-inflammatory condition [40]. Lastly, interpretation was particularly limited for retinol given the low number of data points especially when stratified by age.

In this cohort, estimates of VAD among infants were sharply different by biomarker, cut-off, and adjustment. Differences by biomarker and cut-off highlight the fact that RBP and retinol were not present in a 1:1 molar ratio in this population, which is evidenced by the calculated cut-off of <0.33 µmol/L (as opposed to the retinol cut-off of <0.70 µmol/L). Remaining differences in VAD prevalence when comparing retinol <0.7 µmol/L to RBP <0.33 µmol/L may be explained by the low sample size used to generate this cut-off, which may have been unduly influenced by outliers. These findings underscore the importance of including an adequate retinol sub-sample (at least 10% but preferably more) in any vitamin A study to validate cut-offs. Our data-generated cut-off was very different from others that have been generated and used, which suggests that use of a literature-based cut-off may be insufficient given potential differences in study populations [9,10,22].

In our study of primarily term and normal birthweight infants, we noted a marked improvement in vitamin A status with an increasing infant age. This is consistent with other studies including higher proportions of preterm and low birthweight infants [41,42] as well as a longitudinal study following older, term infants [43]. This trend may reflect the biologic deficit at birth [23] as well as the impact of vitamin A sourced from breastmilk in early infancy and supplementation or consumption of fortified foods as infants age. While patterns among very low birthweight and preterm infants have not been as clear [44,45], it may be that neither RBP nor retinol are good measures of vitamin A status among young, preterm infants [46].

The estimated level of VAD in older infants when using the commonly used cut-off of RBP <0.70 µmol/L was comparable to other countries in the region (in the middle or lower range) regardless of the adjustment for inflammation [3]. The estimated level in mothers was on the lower end of the range seen in the region [3], which may indicate successful implementation of fortification and supplementation in women of reproductive age. For the youngest infants, VAD was high when defined as RBP <0.70 µmol/L or serum retinol <0.70 µmol/L but non-existent when using the calculated RBP cut-off of 0.33 µmol/L. However, early-life low RBP was not significantly associated with morbidity or mortality rates, which suggests that, even if vitamin A status was truly low in this population, it did not have an adverse effect. This is consistent with previous research that failed to find a significant effect of early-life (<6 months of age) VAS on infant morbidities or mortality [27]. It is recognized that very young infants tend to have low vitamin A stores, and it may be that low vitamin A status does not cause ill effects unless it remains low through older infancy and childhood. In the present study, exclusive breastfeeding was significantly associated with VAD at approximately two months of age. Evidence from other populations has been mixed [47,48,49]. This finding may be the result of chance, uncontrolled or residual confounding, or a biological relationship that remains unclear.

This study was conducted among a relatively healthy population exposed to vitamin A through fortified staples, MNPs, and supplementation and the generalizability of our findings should be considered within that context. Even though the population is not representative of Bolivia as a whole, the prevalence of obesity, the level of education, and other sociodemographic markers are in line with urban Bolivian populations especially in this area as per DHS [50]. Our work has several strengths. First, the longitudinal design enabled us to track the vitamin A status among a cohort of healthy, primarily breastfed infants across a full year of life. Furthermore, we also measured CRP and AGP, which allowed us to assess the relationship of inflammation to vitamin A biomarkers and explore the impact of adjusting RBP and retinol for this relationship. Last, the measurement of retinol in addition to RBP permitted the comparison of RBP to retinol and the calculation of a new cut-off that could be applied to RBP and thereby account for a non-1:1 molar relationship between the two vitamin A biomarkers. Nonetheless, the study was limited in that we were able to test only a 10% sample for retinol. Therefore, relationships between retinol and inflammation were harder to quantify precisely especially when samples were stratified by age or the mother-infant status. Furthermore, retinol and RBP are most appropriate for population measurements. A modified relative dose response (MRDR) is best for diagnosing individual VAD but was not performed. However, the purpose of this study was not to assess the effect of vitamin A supplementation, and any VAD misclassification is likely to be non-differential by exposures tested. As in any model, there exists the possibility of residual or unmeasured confounding in our analysis of the effect of early-life VAD on the morbidity and mortality rates as well as in the analysis of predictors of early-life VAD. Furthermore, the mortality analysis was reduced in power due to the rarity of this outcome. Lastly, there is no gold standard for adjusting nutritional biomarkers for inflammation. Our method takes into account the (log) linear relationship between inflammation and RBP and retinol, and previous research has not identified significant interaction [51]. However, there is a possibility that there may be other potential modifiers or confounders of this relationship whose inclusion in the adjustment model would enable better correction of vitamin A biomarkers for the effect of inflammation. For instance, it is possible that vitamin D status, which was not measured but may be low in this high-altitude population could have acted as a confounder of the RBP-CRP relationship [52]. The presence of genetic differences in retinol metabolism could have also potentially obscured the relationships tested [53,54]. Lastly, while the direct effect of inflammation on vitamin A biomarkers is well known, it is possible that there was an additional effect of a low vitamin A status on inflammation (through increased vulnerability to an infectious disease) that was not captured.

## 5. Conclusions

This study confirmed some relationships between inflammation and vitamin A biomarkers but suggested that this relationship may not be fully described by linear regression or other commonly used adjustment methods. The marked difference between the 1:1 molar ratio VAD cut-off (0.7 μmol/L) and the data-generated cut-off (0.33 μmol/L) also underscored the importance of study-specific retinol validation of RBP where resources allow. Lastly, the study suggested that a low vitamin A status in early infancy (~2 months of age) may not have significant effects on morbidity and is likely to improve as infants age and increase their retinol intake via feeding or supplementation.

## Figures and Tables

**Table 1:** Characteristics of the study population.

	*N* or Mean ± SD	Frequency (%)
**Infant Characteristics**		
Male	195	53.4
Caesarian section birth	104	28.5
Preterm (<37 weeks)	67	18.8
Low birth weight (<2500 g)	22	6.5
Birth interval < 36 months ^1^	60	17.0
Ever breastfed	362	99.2
**Maternal Characteristics**		
Age (years)	25.4 ± 6.4	–
Primipara	218	47.9
Overweight or obese (Body Mass Index > 25 kg/m^2^)	225	57.5
Married or cohabiting with partner	395	86.8
Employed	116	25.6
**Maternal Education**		
At least some secondary schooling	99	21.9
Completed secondary school	176	38.9
Incomplete secondary education	178	39.3
**Household Characteristics**		
Number of people in household	5.1 ± 2.1	–
Refrigerator	117	25.7
High-quality flooring ^2^	148	32.5
Water piped inside home	168	36.9
Private toilet	261	57.4

^1^ Versus ≥ 36 months or firstborn. ^2^ Polished wood, parquet, rug, carpet, or tile (as compared to dirt, wooden boards, cement, or brick).

**Table 2:** Time-varying characteristics of the study population *.

	2 Months ^†^		6–8 Months		12–18 Months	
	*N* or Mean ± SD	Frequency (%)	*N* or Mean ± SD	Frequency (%)	*N* or Mean ± SD	Frequency (%)
**Infants and Toddlers**						
*N*	365	–	310	–	168	–
Age	2.1 ± 0.3	–	6.7 ± 0.9	–	14.2 ± 2.3	–
Currently breastfed	357 **	98.3	296	95.5	138	82.1
Received any vitamin A supplementation	0	0	130	41.9	165	98.2
Stunted (length-for-age *Z* scores [LAZ] < −2)	72	19.8	44	14.4	35	21.1
Overweight (weight-for-length Z score [WFL] > 1)	123	33.8	86	28.2	32	19.3
Anemia present ^¥^	261	71.7	232	74.8	137	82.0
Inflammation present ^‡^	10	2.7	65	21.0	27	16.1
**Mothers**						
*N*	455	–	363	–	–	–
Overweight/obese ^£^	279	61.3	198	54.7	–	–
Anemia present ^€^	134	29.5	53	15.1		
Inflammation present ^‡^	160	35.2	65	17.9	–	–

* All singleton infants with at least first blood draw. ** 214 (59%) exclusively breastfed. ^†^ Mothers measured at 1 month postpartum. ^‡^ CRP > 5 mg/L or AGP > 1 g/L. ^¥^ Using altitude-adjusted WHO cutoff.^£^ BMI ≥ 25 kg/m^2^. ^€^ Using altitude-adjusted WHO cutoff (adjusted for smoking in smokers).

**Table 3:** Infant and maternal vitamin A status by biomarker and cut-off at different time points.

	Retinol-Binding Protein (RBP)							Retinol				
Time Point	*N*	Crude			Adjusted ^‡^			*N*	Crude		Adjusted ^‡^	
		Continuous RBP (Mean ± SD)	VAD		Continuous RBP (Mean ± SD)	VAD			Continuous Retinol (Mean ± SD)	VAD	Continuous Retinol (Mean ± SD)	VAD
**Infants**			**<0.33 μmol/L**	**<0.7 μmol/L**		**<0.33 μmol/L**	**<0.7 μmol/L**			**<0.7 μmol/L**		**<0.7 μmol/L**
2 Months	365	0.63 ± 0.15	0 (0%)	259 (71.0%)	–	–	–	38	0.95 ± 0.24	7 (18.4%)	–	–
6–8 Months	310	0.84 ± 0.22	0 (0%)	88 (28.4%)	0.91 ± 0.23	0 (0%)	46 (14.8%)	29	1.21 ± 0.33	2 (6.9%)	1.39 ± 0.37	0 (0%)
12–18 Months	168	0.90 ± 0.23	0 (0%)	26 (15.5%)	0.97 ± 0.24	0 (0%)	13 (7.7%)	9	1.27 ± 0.37	0 (0%)	1.46 ± 0.34	0 (0%)
**Mothers**			**<0.65 μmol/L**	**<0.7 μmol/L**		**<0.65 μmol/L**	**<0.7 μmol/L**			**<0.7 μmol/L**		**<0.7 μmol/L**
1 Month postpartum	455	1.73 ± 0.54	3 (0.7%)	4 (0.9%)	1.83 ± 0.56	3 (0.7%)	3 (0.7%)	45	1.88 ± 0.46	0 (0%)	2.04 ± 0.49	0 (0%)
6–8 Months postpartum	363	1.62 ± 0.44	1 (0.3%)	1 (0.3%)	1.69 ± 0.44	1 (0.3%)	1 (0.3%)	37	1.96 ± 0.36	0 (0%)	2.09 ± 0.39	0 (0%)

VAD: Vitamin A Deficiency. Cut-offs employed are indicated in boldface. ^‡^ Adjusted using linear regression with coefficients derived from data pooled across second and third blood draws for infants and first and second blood draws for mothers. First blood draw values are not adjusted for infants given the observed lack of correlation between inflammatory biomarkers and vitamin A biomarkers.

**Table 4:** Effects of early vitamin A deficiency (VAD; RBP < 0.70 µmol/L) on rates of reported morbidity episodes (*N* = 330) *.

	Crude			Adjusted **		
	Estimate	95% CI	*p*-Value ^†^	Estimate	95% CI	*p*-Value ^†^
**Rates (Morbidity episodes per 100 child-months)**						
Overall population	7.89	(7.41, 8.39)	–	–	–	–
Non-VAD at 2 months	8.22	(7.35, 9.19)	–	8.65	(7.01, 10.68)	–
VAD at 2 months	7.75	(7.20, 8.35)	–	8.13	(6.78, 9.76)	–
Rate Ratio	0.94	(0.82, 1.08)	0.39	0.94	(0.82, 1.09)	0.41

* Morbidity episodes included fever, respiratory illness/coughing, or diarrhea. ** Adjusted for maternal employment, low birth weight (<2500 g), and inflammation-adjusted ferritin. ^†^ Wald Chi Square.

## Abbreviations

VAD	(vitamin A deficiency)
RBP	(retinol-binding protein)
CRP	(C-reactive protein)
AGP	(alpha(1)-acid glycoprotein)
MNP	(multiple micronutrient powder)
VAS	(vitamin A supplementation)
